# Clinical outcomes after ventricular tachycardia ablation with or without induction

**DOI:** 10.1016/j.hroo.2024.10.023

**Published:** 2024-11-17

**Authors:** Benjamin L. Freedman, Shu Yang, Jonathan W. Waks, Andrew Locke, Timothy R. Maher, Andre d’Avila

**Affiliations:** 1 Division of Cardiology, Tufts Medical Center, Boston, Massachusetts; 2 Harvard-Thorndike Electrophysiology Institute and Arrhythmia Service, Division of Cardiovascular Medicine, Department of Medicine, Beth Israel Deaconess Medical Center, Harvard Medical School, Boston, Massachusetts

**Keywords:** Catheter ablation, Ventricular tachycardia, Ventricular tachycardia ablation, Ventricular tachycardia induction, Hemodynamic instability, Substrate modification, Ventricular tachycardia mapping

## Abstract

**Background:**

Substrate mapping may offer a safer alternative to ventricular tachycardia (VT) mapping by avoiding prolonged episodes of VT during catheter ablation. However, VT induction to gauge procedural efficacy is still routinely attempted following substrate ablation, thereby exposing patients to potentially unnecessary hemodynamic risk.

**Objective:**

The purpose of this study was to assess the efficacy of VT ablation without VT induction.

**Methods:**

Patients with implantable cardioverter-defibrillators who underwent VT ablation between August 2020 and May 2023 were assessed retrospectively. Ablation and induction strategies were determined by operator discretion. Patients with or without attempted VT induction were compared with respect to baseline characteristics and clinical outcomes using Cox and competing risks regression.

**Results:**

Eighty-nine patients (median age 68 years; 89% male; 51% infarct-related cardiomyopathy, mean left ventricular ejection fraction 38%) were followed for a median of 16 months after VT ablation. VT induction was attempted in 63% of patients. The 1-year incidence of recurrent VT was 37% and 58% in the noninduction and induction groups, respectively (subhazard ratio 0.55, 95% confidence interval [CI] 0.27–1.09, *P* = .09). The 1-year incidence of recurrent VT, heart transplant, or death was 42% and 62% in the noninduction and induction groups, respectively (hazard ratio 0.58, 95% CI 0.31–1.11, *P* = .10).

**Conclusion:**

In a single-center study of 89 VT ablations, a noninduction strategy was similar to an induction strategy with respect to VT recurrence, heart transplant, or death at 1 year. Our findings suggest that VT induction, recognized as a risk factor for hemodynamic compromise, can be avoided in some patients without sacrificing procedural efficacy.

**Key Findings**

▪ In a retrospective study of 89 patients with an implantable cardioverter-defibrillator undergoing ventricular tachycardia (VT) ablation, a strategy of avoiding VT induction vs attempting VT induction resulted in a similar incidence of VT recurrence, heart transplant, or death at 1 year.
▪ Both ablation strategies were associated with >95% median reduction in VT burden over a 6-month period.
▪ Our findings suggest that VT induction, recognized as a risk factor for hemodynamic compromise, may be avoided in some patients without sacrificing procedural efficacy.

## Introduction

Ventricular tachycardia (VT) is a significant cause of morbidity and mortality in the United States, particularly as the prevalence of heart failure increases in an aging population.^1^ Although implantable cardioverter-defibrillators (ICDs) are instrumental in reducing mortality from sudden cardiac arrest in patients with previous VT, ICD shocks themselves impair quality of life and may precipitate worsening heart failure.^2^^,^^3^ Catheter ablation for VT is an important synergistic therapy and has been shown to reduce the incidence of appropriate ICD shocks and hospitalizations for cardiovascular causes.^4^, ^5^, ^6^

Undesirable hemodynamic decompensation frequently occurs during VT ablation and has been associated with higher rates of in-hospital acute kidney injury (AKI), as well as higher mortality at 1 month and 1 year postablation.^7^^,^^8^ Because of that, several risk factors for intraprocedural hemodynamic compromise (chronic obstructive pulmonary disease, age >60 years, general anesthesia, ischemic cardiomyopathy, New York Heart Association (NYHA) functional class III or IV, LVEF <25%, VT storm, diabetes [PAINESD score]), have been identified to predict which patients will benefit from percutaneous left ventricular assist device (LVAD) support during ablation.^9^^,^^10^

Alternatively, newer VT ablation techniques, collectively termed “substrate modification,” have been developed with the goal of identifying VT critical sites by mapping during sinus or paced rhythms rather than during VT. Many substrate modification techniques target abnormalities in the static components of local bipolar electrograms, for example, signal amplitude (voltage mapping), morphology (local abnormal ventricular activity), or timing (late potentials). Others, such as isochronal late activation mapping, focus on dynamic/functional properties of the myocardium, targeting regions of maximal conduction slowing.^11^^,^^12^

In general, substrate modification has shown equivalent or superior efficacy and safety compared to activation and entrainment mapping, which supports the hypothesis that minimizing time in VT during ablation is beneficial.^13^, ^14^, ^15^ However, even when substrate modification is performed, it remains common practice to attempt VT induction before and after ablation in hopes of demonstrating postablation noninducibility, traditionally considered to be a metric of procedural success. In this single-center observational cohort study, we sought to determine the clinical impact of a VT noninduction strategy by assessing short-term safety outcomes (eg, 30-day mortality) as well as long-term efficacy outcomes (eg, time to first VT recurrence) in patients undergoing VT ablation with or without attempted VT induction. We hypothesized that by minimizing intraprocedural hemodynamic instability, a noninduction strategy would be associated with improved short-term safety without sacrificing the long-term effectiveness of VT ablation.

## Methods

### Patient selection

One hundred four consecutive patients with ≥1 sustained VT episode who underwent VT ablation at Beth Israel Deaconess Medical Center between August 2020 and May 2023 were screened retrospectively. Patients were excluded if they had a durable LVAD, if they had no ICD in the postablation follow-up period, or if they underwent an electrophysiological study but had no ventricular ablation lesions delivered. For patients who underwent >1 VT ablation during the follow-up period, only the first VT ablation (referred to as “initial ablation”) was analyzed for clinical outcomes. The research reported in this paper adhered to the Helsinki Declaration and was approved by the Institutional Review Board of Beth Israel Deaconess Medical Center.

### Comparator groups

In our primary analysis, we compared the outcomes of 2 ablation strategies: no attempted VT induction or any attempted VT induction (referred to as “noninduction” and “induction” strategies). The induction strategy encompassed attempts to induce VT at any time during the procedure (Supplemental Table 1) with programmed electrical stimulation, for any purpose (eg, to perform activation or entrainment mapping, to obtain a 12-lead rhythm strip for pacemapping, or to assess postablation inducibility). As a reflection of real-world practice, the induction group included some patients with unsuccessful induction attempts who therefore had no intraprocedural VT, and the noninduction group included some patients who experienced spontaneous VT during the ablation initiated by ectopic beats. In sensitivity analyses, we also compared clinical outcomes by (1) the presence or absence of intraprocedural sustained VT, regardless of induction strategy; and (2) the presence or absence of postablation inducibility testing (ie, attempted VT induction at the end of the procedure).

### Clinical outcomes

Clinical outcomes were ascertained by review of the electronic medical record. Long-term efficacy outcomes included mortality, heart transplant, recurrent sustained VT, and change in VT burden. Short-term safety outcomes included immediate postprocedural complications, AKI, transfer to the cardiac intensive care unit (CCU), time to discharge, death or rehospitalization within 30 days, and change in left ventricular ejection fraction (LVEF). Sustained VT was defined as any VT or ventricular fibrillation episode lasting ≥30 seconds or resulting in ICD shock or antitachycardia pacing. VT episodes were verified by review of ICD interrogation electrograms. Baseline serum creatinine was defined as the most recent value documented within 1 year preceding ablation. AKI was defined as an increase in serum creatinine by ≥0.3 mg/dL within 48 hours or by 50% within 7 days.^16^ To assess the change in VT burden, the overall number of sustained VT episodes was quantified in the 6-month period before and after VT ablation. Patients who died within 6 months after ablation or lacked an ICD in the 6 months preceding VT ablation were excluded from the VT burden analysis. Baseline LVEF was obtained from the most recent echocardiogram or cardiac magnetic resonance imaging within 1 year preceding VT ablation. Postablation change in LVEF was analyzed in patients who underwent echocardiography or cardiac magnetic resonance imaging within 3 months before and after ablation. Patients who died during their index hospitalization were excluded from analyses of time to discharge and 30-day rehospitalization rate. Patients located in the CCU before VT ablation were excluded from the analysis of postablation CCU transfer.

### Mapping and catheter ablation of VT

Electroanatomic mapping was performed using CARTO 3 Version 7 (Biosense Webster, Irvine, CA) or EnSite Precision or Ensite X (Abbott Laboratories, Green Oaks, IL). Catheter ablation of VT was performed as previously described.^12^^,^^17^ After obtaining informed consent, patients were taken to the electrophysiology laboratory in a fasting state and placed under general anesthesia. All patients underwent activation mapping during either sinus/atrial-paced rhythm, right ventricular pacing, and/or lateral left ventricular pacing (via a coronary sinus branch pacing lead) at the operator’s discretion. For late activation mapping, local activation time was annotated at the latest sharp bipolar electrogram with corresponding unipolar deflection. If there was no late unipolar deflection, the last bipolar deflection above the visual noise reproducible on consecutive beats was annotated.

The specific ablation strategy varied by patient at the operator’s discretion and targeted a combination of (1) wavefront discontinuity lines (ie, an automated representation of isochronal crowding^12^; (2) sites of best pacemap matches with a clinical VT; and/or (3) areas identified as critical sites during VT entrainment or activation mapping of spontaneous or induced VT. In order to minimize the risk of intraprocedural hemodynamic decompensation, programmed electrical stimulation was not routinely attempted in patients with known hemodynamically unstable VT, pacing-induced hypotension, or abnormal functional substrate identified by late activation mapping, at the operator’s discretion. Anesthesia was always administered before attempted VT induction. Ablation was performed using a 3.5-mm-tip, open-irrigated ablation catheter (Biosense Webster Thermocool Smarttouch SF or Abbott TactiCath Sensor Enabled) at 30–45 W titrated to a current between 650 and 720 mA for 60–120 seconds using a contact force targeted between 8g and 25g.

### Statistical analysis

Unless otherwise specified, continuous variables are given as mean ± SD when normally distributed and median (25th percentile, 75th percentile [P_25_, P_75_]) when non-normally distributed. Categorical variables are given as number (%) of patients, rounded to the nearest integer. The cumulative incidence of first VT recurrence and of VT recurrence, heart transplant, or death are reported at 1 year of follow-up and were derived using the Kaplan-Meier method. Univariable and multivariable predictors of VT recurrence, transplant, or death were identified using Cox proportional hazards regression. Competing risks regression was used to identify predictors of first VT recurrence while controlling for the competing risks of death or heart transplant. Baseline characteristics were included in multivariable models for the primary analysis only if their univariable association with the outcome of interest had P <.05. To mitigate the potential for confounding by heterogeneous ablation strategies, the use of any VT mapping and the use of any substrate modification were included (regardless of univariable P value) in multivariable models in a sensitivity analysis. All other clinical outcomes were compared using the Pearson χ^2^ test (or Fisher exact test where appropriate) for categorical variables and the Wilcoxon rank sum test for continuous variables (all of which were non-normally distributed). Statistical significance was defined as P <.05 in all cases. No adjustment was made for multiple comparisons. Statistical analyses were performed using STATA Version 16.1 (StataCorp, College Park, TX).

## Results

### Baseline and procedural characteristics

Between August 2020 and May 2023, 104 patients underwent ≥1 attempted VT ablation at Beth Israel Deaconess Medical Center. Of these patients, 15 were excluded from the analysis—1 had an LVAD, 4 had no ICD during postablation follow-up, and 10 had mapping but no ablation performed (Supplemental Figure 1). Thus, a total of 89 patients were included in the study: 33 (37%) in the noninduction group and 56 (63%) in the induction group. Median follow-up after initial ablation was 16 months. Thirty-nine of 56 patients (70%) in the induction group had VT induced successfully. The prevalence of spontaneous VT was statistically similar between the noninduction (45%) and induction (64%) groups (*P* = .08). As such, one or more episodes of induced or spontaneous VT occurred in 45% of ablations in the noninduction group compared to 82% of ablations in the induction group (*P* <.001). Hemodynamically unstable VT occurred less frequently in the noninduction group than in the induction group (24% vs 54%, *P* = .007) (Supplemental Table 1).

Patient baseline characteristics are summarized in Table 1. Median age was 68 [61, 74] years, and 89% of patients were male. Underlying structural heart disease was present in all patients: infarct-related cardiomyopathy (ICM) in 51%, non–infarct-related cardiomyopathy (NICM) in 46%, and hypertrophic cardiomyopathy in 3%. Comorbid atrial fibrillation or flutter was present in 42% of patients. Mean baseline LVEF was 38% ± 13%, and median baseline serum creatinine was 1.1 [0.9, 1.4] mg/dL. One-fourth of patients had diabetes mellitus, and one-third presented in VT storm. The median PAINESD score was 10 [4, 14], with 23% of patients having a PAINESD score ≥15, corresponding to a high risk for periprocedural hemodynamic compromise (Supplemental Figure 2). Nineteen percent of patients had undergone VT ablation before the study period, and 80% of patients were taking ≥1 antiarrhythmic medication at the time of ablation. Differences between the noninduction and induction groups were minor with the exception of NYHA class III–IV heart failure symptoms (9% vs 27%) and comorbid chronic obstructive pulmonary disease (12% vs 25%), both of which were more common in the induction group.

**Table 1:** Baseline characteristics

Baseline characteristics	All patients (N = 89)	Noninduction (n = 33)	Induction (n = 56)	*P* value
Age, y	68 [61, 74]	65 [62, 70]	69 [61, 76]	.26
Male sex	79 (89)	32 (97)	47 (84)	.06
Cardiomyopathy type				.72
Infarct-related	45 (51)	19 (58)	26 (46)	
Hypertrophic	3 (3)	1 (3)	2 (4)	
Other non–infarct-related	41 (46)	13 (39)	28 (50)	
Atrial fibrillation or flutter	37 (42)	15 (45)	22 (39)	.57
Baseline LVEF, %	38 ± 13	35 ± 14	39 ± 13	.23
NYHA functional class III–IV heart failure	18 (20)	3 (9)	15 (27)	.04
COPD	18 (20)	4 (12)	14 (25)	.14
Serum creatinine, mg/dL	1.1 [0.9, 1.4]	1.1 [0.9, 1.4]	1.1 [0.9, 1.5]	.5
Diabetes	22 (25)	10 (30)	12 (21)	.35
VT storm	29 (33)	12 (36)	17 (30)	.56
PAINESD score	10 [4, 14]	12 [6, 14]	9 [3, 15]	.53
Previous VT ablation	17 (19)	7 (21)	10 (18)	.70
Antiarrhythmic medication	71 (80)	27 (82)	44 (79)	.71

Values are given as median [25th percentile, 75th percentile], n (%), or mean ± SD unless otherwise indicated.

New York Heart Association (NYHA) functional class (and therefore PAINESD score) was unavailable for 1 patient in the induction group. Baseline serum creatinine was unavailable in 2 patients in the noninduction group and 3 patients in the induction group.

COPD = chronic obstructive pulmonary disease; LVEF = left ventricular ejection fraction; VT = ventricular tachycardia.

Ablation characteristics are given in Table 2. Preablation cardiac computed tomographic angiography or cardiac magnetic resonance imaging performed in 57% of patients. Median procedural time was 206 [170, 251] minutes. Mean number of ablation lesions was 35 ± 17, and mean total radiofrequency time per patient was 1725 ± 885 seconds. Twenty percent of ablations involved epicardial access. The latter characteristics were proportionally similar between groups. In contrast, outpatient ablations were less common in the noninduction group (38% vs 61%), as was intraprocedural use of a percutaneous LVAD (4% vs 12%). Some patients in the noninduction group had activation/entrainment mapping performed during spontaneous VT, whereas some patients in the induction group were noninducible and underwent exclusively substrate modification. An ablation strategy of substate modification without VT activation or entrainment mapping was used in 60% of patients overall and was more common in the noninduction group (88% vs 43%). An ablation strategy based on VT activation and entrainment mapping without substrate modification was used in 13% of patients overall and was more common in the induction group (20% vs 3%). A combination strategy including both substrate modification and VT mapping was used in 26% of patients overall and was more common in the induction group (36% vs 9%).

**Table 2:** Procedural characteristics

Procedural characteristics	All patients (N = 89)	Noninduction (n = 33)	Induction (n = 56)	*P* value
Outpatient ablation	41 (46)	20 (61)	21 (38)	.04
Preablation CT/MRI	51 (57)	20 (61)	31 (55)	.63
Procedural time,∗ min	206 [170, 251]	212 [181, 276]	200 [162, 237]	.15
Radiofrequency time,† s	1725 ± 885	1951 ± 887	1587 ± 868	.16
Ablation lesions‡	35 ± 17	38 ± 17	32 ± 16	.22
Epicardial access	18 (20)	8 (24)	10 (18)	.47
pLVAD support	6 (7)	4 (12)	2 (4)	.19
Mapping strategy				<.001
Substrate and VT mapping	23 (26)	3 (9)	20 (36)	
Substrate mapping only	53 (60)	29 (88)	24 (43)	
VT mapping only	12 (13)	1 (3)	11 (20)	

Values are given as n (%), median [25th percentile, 75th percentile], or mean ± SD unless otherwise indicated.

CT = computed tomography; MRI = magnetic resonance imaging; pLVAD = percutaneous left ventricular assist device; VT = ventricular tachycardia.

∗ Procedural time was unavailable in 1 patient in the noninduction group.

† Radiofrequency time was unavailable in 14 patients in the noninduction group and 25 patients in the induction group.

‡ Number of ablation lesions was unavailable in 11 patients in the noninduction group and 22 patients in the induction group.

### Clinical outcomes and predictors

**Figure 1:**
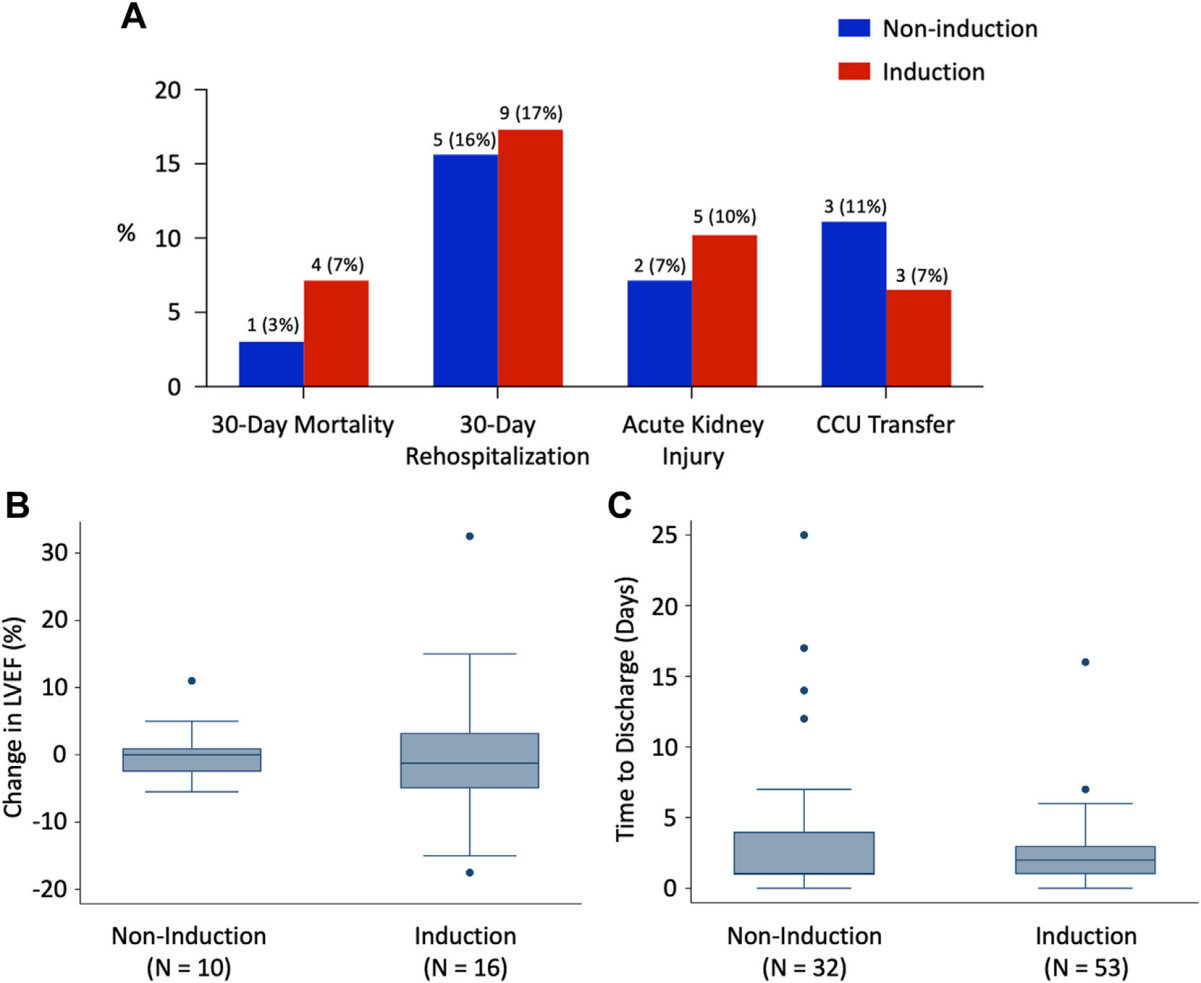
Short-term safety outcomes. Data were unavailable in the noninduction and induction groups, respectively, in 1 and 4 patients for 30-day rehospitalization, 5 and 7 patients for acute kidney injury, 6 and 10 patients for cardiac intensive care unit transfer, 23 and 40 patients for change in left ventricular ejection fraction, and 1 and 3 patients for time to discharge. **A:** Bar graph of categorical safety outcomes, labeled with the number (%) of patients experiencing each outcome. **B, C:** Box plots of continuous safety outcomes. The median is depicted by the central line of each box. The lower and upper edges of the box represent the 25th and 75th percentiles. The lower and upper whiskers correspond to P_25_ – (1.5 ∗ IQR) and P_75_ + (1.5 ∗ IQR). *Dots* represent potential outliers. Change in LVEF is expressed as an absolute difference. There were no significant differences between groups for any outcome. IQR = interquartile range.

Short-term safety outcomes are shown in Figure 1 and did not differ significantly between induction groups. A total of 5 patients (6%) died within 30 days after VT ablation. Fourteen patients (17%) required rehospitalization within 30 days: 5 for recurrent VT, 3 for other cardiac causes, and 6 for noncardiac causes. Seven patients (9%) experienced AKI, and 6 (8%) required transfer to the CCU after ablation—3 for hypotension, 2 for acute hypoxemic respiratory failure, and 1 for frequent nonsustained VT. Median change in LVEF was –0.5% [–5%, 2.5%] and median time to hospital discharge was 2 [1, 4] days. Thirteen patients (15%) experienced a range of other acute procedural complications, including 2 patients with high-grade atrioventricular block, 4 with respiratory failure (ie, inability to be extubated in the postanesthesia care unit), 1 with postprocedure hypotension requiring pressor support, 3 with pericarditis, 1 cardioembolic stroke, 1 transient ischemic attack, 1 cardiac tamponade, and 1 case of methicillin-resistant *Staphylococcus aureus* bacteremia presenting 2.5 weeks after ablation (Supplemental Table 2).

**Figure 2:**
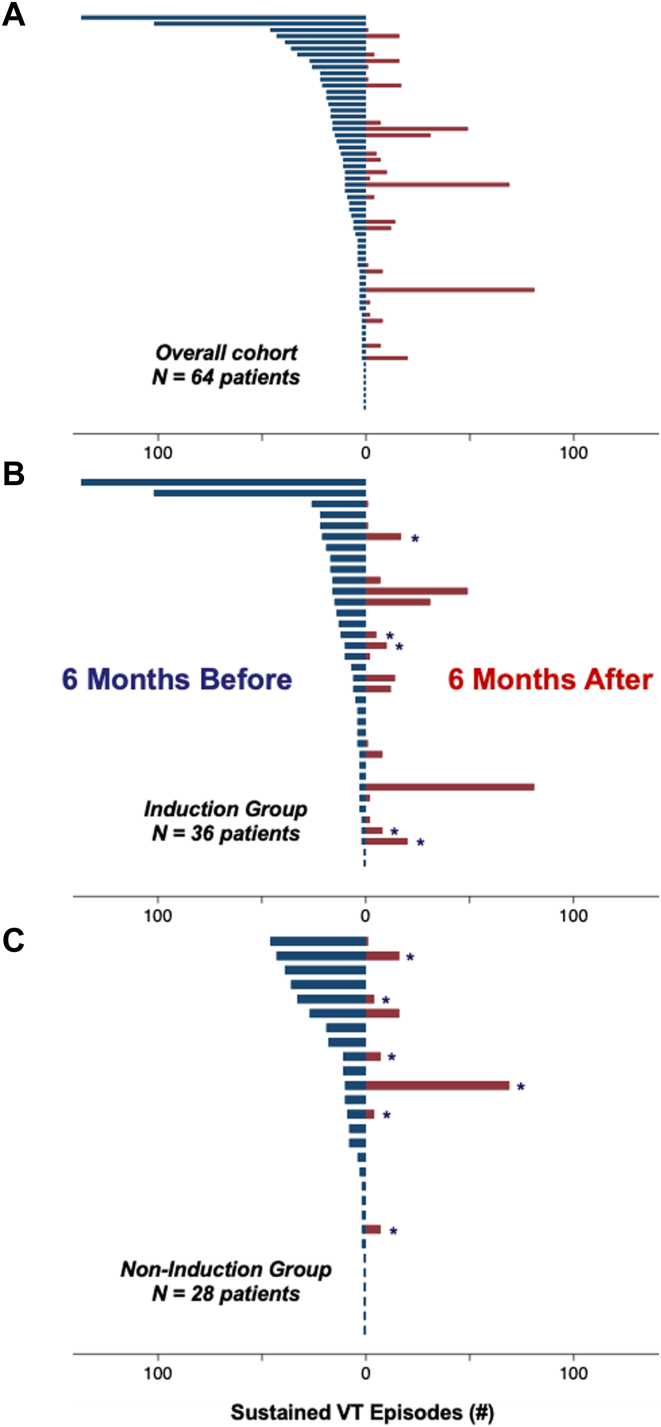
Ventricular tachycardia (VT) burden before vs after ablation. Each patient is represented by a *horizontal bar.* VT burden in the 6 months preceding ablation is shown in *blue* to the left of the midline. VT burden in the 6 months following ablation is shown in *red* to the right of the midline. Fifteen patients from the induction group were excluded from the VT burden analysis: 7 who died within 6 months after ablation and 8 who lacked an implantable cardioverter-defibrillator (ICD) in the 6 months preceding ablation. Five patients in the noninduction group were excluded from the VT burden analysis: 3 who died within 6 months after ablation and 2 who lacked an ICD in the 6 months preceding ablation. A marked decrease in VT burden is seen following ablation in the overall patient cohort **(A),** as well as the VT induction **(B)** and noninduction **(C)** groups. Eleven patients (5 in the induction group, 6 in the noninduction group) underwent repeat VT ablation within the 6-month period following their first ablation. These patients are denoted by an *asterisk* in plots B and C.

**Figure 3:**
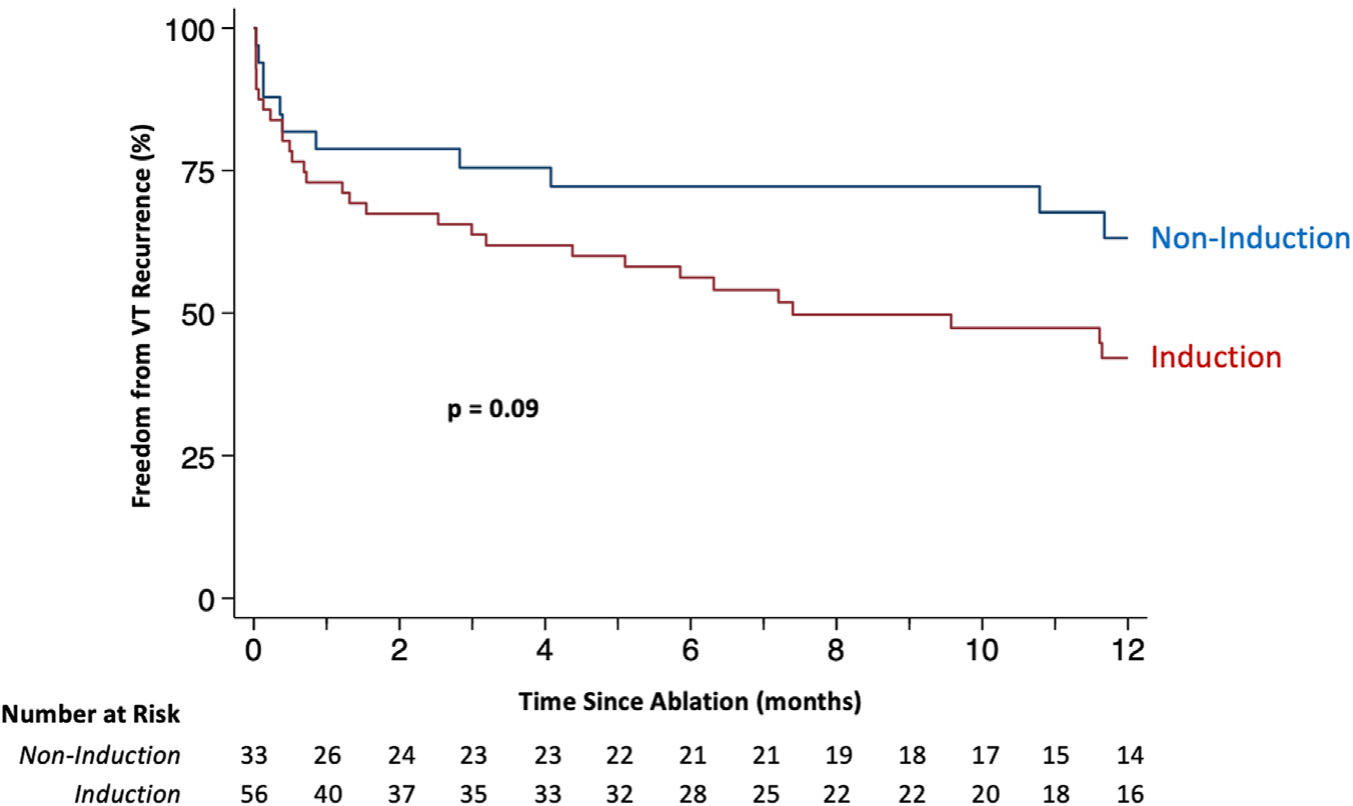
Ventricular tachycardia (VT) recurrence stratified by induction strategy. The cumulative probability of freedom from first recurrent sustained VT is plotted as a function of time elapsed since VT ablation. *P* value for noninduction vs induction groups is derived from a univariable competing risks regression of VT recurrence.

**Figure 4:**
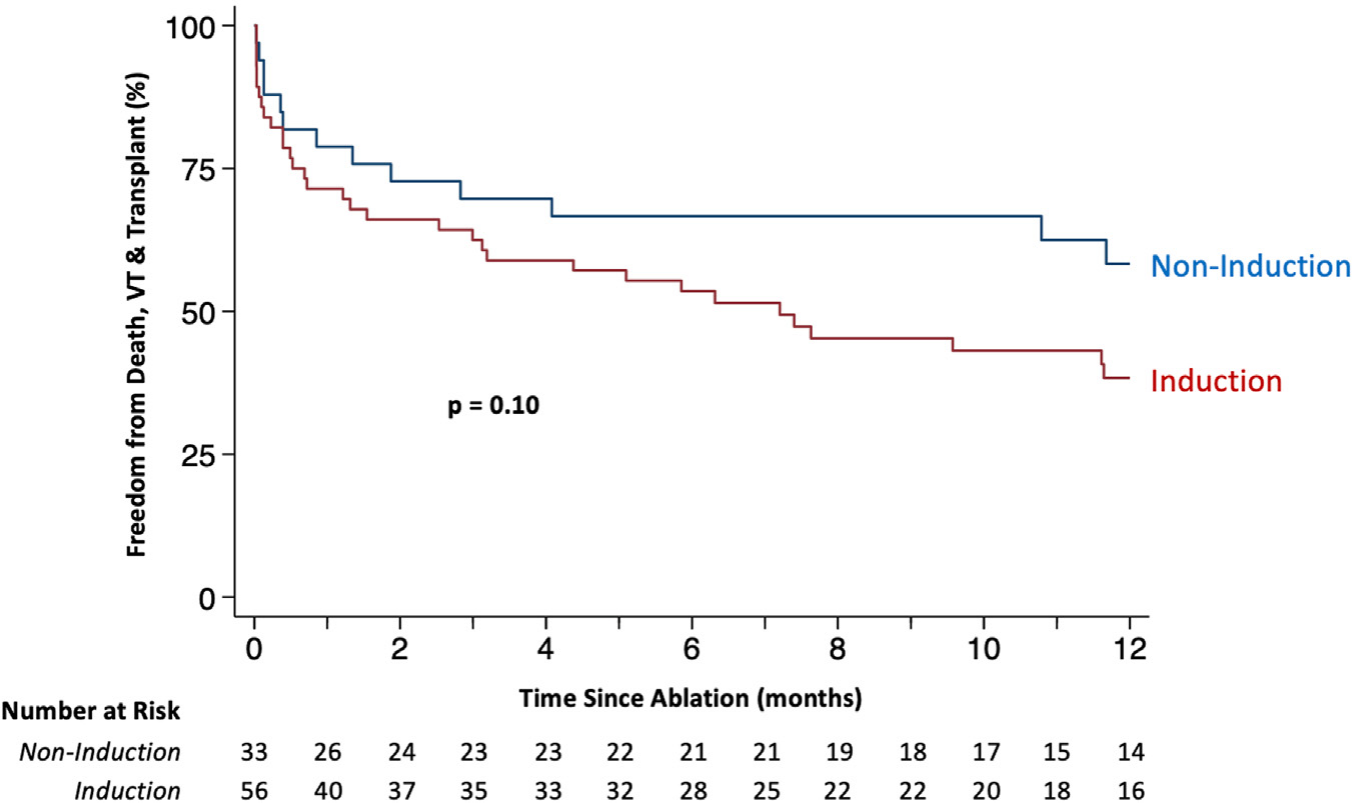
Ventricular tachycardia (VT) recurrence, heart transplant, or death Stratified by induction strategy. The cumulative probability of freedom from VT recurrence, heart transplant, and death is plotted as a function of time elapsed since VT ablation. *P* value for noninduction vs induction groups is derived from a univariable Cox regression of VT recurrence, heart transplant, or death.

Six-month VT burden was reduced by a median of 100% [93%, 100%] in the noninduction group and 96% [0%, 100%] in the induction group (*P* = .046) (Figure 2). At 1 year of follow-up, VT recurred in 11 patients (37%) in the noninduction group and 30 patients (58%) in the induction group (subhazard ratio [SHR] 0.55, 95% confidence interval [CI] 0.27–1.09, *P* = .09) (Table 3 and Figure 3). VT recurred in 29 patients (48%) without postablation inducibility testing and 12 patients (58%) with postablation inducibility testing (SHR 0.74, 95% CI 0.37–1.45, *P* = .37) (Figure 3 and Supplemental Figure 3). There were 4 deaths and no heart transplants in the noninduction group. There was 1 heart transplant and 10 deaths in the induction group, 1 of which occurred after heart transplant. The composite of VT recurrence, heart transplant, or death occurred in 13 patients (42%) in the noninduction group and in 33 patients (62%) in the induction group (hazard ratio [HR] 0.58, 95% CI 0.31–1.11, *P* = 0.10) (Figure 4 and Table 4). Sensitivity analysis comparing patients with or without intraprocedural sustained VT (regardless of induction strategy) revealed no differences in short-term safety outcomes or long-term clinical outcomes (Supplemental Table 3).

**Table 3:** Competing risks regression of VT recurrence

Clinical characteristics	Univariable	
	SHR (95% CI)	*P* value
Outpatient ablation	0.61 (0.32–1.14)	.12
Six-month preablation VT burden (per 5-episode increase)	1.01 (0.98–1.06)	.47
Age (per 10-year increase)	1.27 (0.91–1.77)	.16
Sex (male vs female)	1.42 (0.55–.3.62)	.47
COPD	1.07 (0.52–2.18)	.86
Diabetes	1.21 (0.61–2.41)	.59
Baseline Cr	1.24 (0.86–1.79)	.25
Cardiomyopathy (NICM vs ICM)	**1.90 (1.02–3.54)**	**.04**
NYHA functional class III–IV heart failure	1.48 (0.72–3.06)	.29
VT mapping performed	1.45 (0.57–3.71)	.44
Substrate modification performed	0.68 (0.28–1.62)	.38
LVEF (per 10% increase)	1.03 (0.79–1.35)	.82
VT storm	1.72 (0.93–3.17)	.08
Previous VT ablation	1.01 (0.47–2.18)	.97
Cardiac CT or MRI	0.56 (0.30–1.02)	.06
Noninduction strategy	0.55 (0.27,1.09)	.09
Absence of intraprocedural VT	0.77 (0.40–1.49)	.44
No postablation inducibility test	0.74 (0.37–1.45)	.37

Death and heart transplant were considered competing risks. Significant associations are denoted by bold text.

CI = confidence interval; Cr = creatinine; ICM = infarct-related cardiomyopathy; NICM = non–infarct-related cardiomyopathy; SHR = subhazard ratio; other abbreviations as in Tables 1 and 2.

**Table 4:** Cox regression of VT recurrence, death, or heart transplant

Clinical characteristics	Univariable		Multivariable	
	HR (95% CI)	*P* value	HR (95% CI)	*P* value
Six-month preablation VT burden (per 5-episode increase)	**1.05 (1.01–1.10)**	**.02**	1.02 (0.96–1.09)	.53
Age (per 10-year increase)	**1.41 (1.02–1.96)**	**.04**	1.42 (0.93–2.18)	.10
Sex (male vs female)	1.69 (0.61–4.71)	.32		
COPD	1.41 (0.73–2.73)	.31		
Diabetes	1.58 (0.84–2.97)	.15		
Baseline Cr	**1.38 (1.07–1.79)**	**.01**	1.05 (0.74–1.49)	.78
Cardiomyopathy (NICM vs ICM)	1.76 (0.98–3.18)	.06		
NYHA functional class III–IV heart failure	**2.46 (1.30–4.66)**	**.005**	1.59 (0.73–1.49)	.24
LVEF (per 10% increase)	0.96 (0.77–1.21)	.76		
VT storm	**2.00 (1.11–3.59)**	**.02**	1.40 (0.64–3.09)	.40
PAINESD score (per 1-point increase)	1.03 (0.99–1.07)	.13		
Previous VT Ablation	1.20 (0.59–2.42)	.61		
Cardiac CT or MRI	0.58 (0.32–1.03)	.06		
Noninduction strategy	0.58 (0.31–1.11)	.10		
Absence of intraprocedural VT	0.62 (0.32–1.19)	.15		
VT mapping performed	1.33 (0.56–3.14)	.52		
Substrate mapping performed	0.82 (0.36–1.82)	.62		
Unstable VT present	1.24 (0.69–2.23)	.47		
No. of unstable VTs (per 1-episode increase)	1.05 (0.82–1.34)	.70		

Significant associations are denoted by bold text.

CI = confidence interval; HR = hazard ratio; other abbreviations as in Tables 1, 2, and 3.

Among all baseline and procedural characteristics (including induction strategy), only cardiomyopathy type was found to be associated with VT recurrence in a univariable competing risks regression (SHR 1.90 for NICM vs ICM, 95% CI 1.02–3.54, *P* = .04) (Table 3). In a multivariable competing risks model adjusting for cardiomyopathy type and the use of substrate modification or VT mapping, induction strategy remained unassociated with VT recurrence (SHR 0.63 for noninduction vs induction, 95% CI 0.30–1.31, *P* = .21) (Supplemental Table 4). Although preablation VT burden, age, baseline creatinine, NYHA class III–IV heart failure, and preablation VT storm all showed univariable associations with the composite of VT recurrence, death, or transplant, none remained significant after multivariable adjustment (Table 4). Furthermore, induction strategy remained unassociated with VT recurrence, death or transplant after multivariable adjustment for NYHA class III–IV Heart Failure and the use of substrate modification or VT mapping in a sensitivity analysis (HR 0.64 for non-induction vs. induction, 95% CI 0.32-1.26, p = 0.19) (Supplemental Table 5).

## Discussion

In this retrospective study of 89 patients with structural heart disease who underwent catheter ablation for VT, we found that a VT noninduction strategy was statistically similar to a VT induction strategy with respect to the 1-year risk of VT recurrence or the composite of VT recurrence, heart transplant, or death. This finding is significant because it suggests that for patients whose VT is known or suspected to be hemodynamically unstable (or whose comorbidities place them at high risk for hemodynamic decompensation [eg, PAINESD score ≥15]), VT induction during ablation can be avoided without sacrificing long-term procedural efficacy. We also found that 6-month VT burden reduction was nominally greater with a noninduction strategy. This finding may represent type I error, given our relatively small sample size for the VT burden analysis (N = 64), the fact that the median VT burden reduction was 100% in both groups, and the lack of a clear explanation for how VT induction would cause an excess of postablation VT.

We previously summarized the potentially deleterious impact of unstable VT on patients’ short- and long-term prognosis following VT ablation.^18^ Temporary mechanical circulatory support may not completely eliminate the risk of hemodynamic decompensation. Miller et al^19^ showed that in 20 patients with scar-related VT and LVEF ≤40% undergoing ablation with noninvasive cerebral oximetry monitoring, 17% of intraprocedural VT episodes caused cerebral desaturation to <55% (requiring pace termination or electrical cardioversion) despite hemodynamic support with the Impella 2.5 percutaneous LVAD and intravenous phenylephrine. In a retrospective study of 317 patients undergoing ablation for scar-related VT, Kuo et al^7^ found that intraprocedural hemodynamic decompensation was independently associated with AKI (OR 3.98, *P* = .03), 30-day mortality (HR 5.13, *P* = .02), and 1-year mortality (HR 2.59, *P* = .05). Santangeli et al^8^ found the presence of unmappable VTs during VT ablation to be an independent predictor of 30-day mortality (OR 5.69, p = 0.017) among 2061 patients in the International VT Ablation Center Collaborative Group database. (Although unmappable VT was not explicitly defined in the latter study, hemodynamic instability is the most common obstacle to VT mapping.^20^)

In contrast, in our study, attempted VT induction did not correspond to a higher risk of subsequent AKI, CCU transfer, prolonged hospital stay, decreased LVEF, or rehospitalization or death at 30 days. However, this should be understood in the context of our relatively small cohort size and correspondingly low event rates (eg, 5 deaths within 30 days and 7 AKIs). Second, it is possible that a noninduction approach was used more often in patients who were referred for very fast or hemodynamically unstable VT, which may have biased our findings toward the null hypothesis of no difference between groups. Third, although several patients during their ablations experienced transient hemodynamic instability during induced or spontaneous VT, we did not have any cases meeting Kuo et al’s definition of acute hemodynamic decompensation (ie, prolonged hypotension requiring urgent mechanical circulatory support or termination of the procedure).^7^ Based on existing evidence from larger cohorts, it is reasonable to speculate that with a larger population size, we might have observed a protective effect of VT noninduction with respect to the aforementioned safety outcomes.

The past 2–3 decades have witnessed the development of several techniques of substrate modification, in part to address the problem of hemodynamic instability during conventional VT activation and entrainment mapping. Although VT induction and mapping once were considered the gold standard method of locating and ablating VT critical sites, this paradigm is shifting because of recent studies demonstrating the equivalent (and in some cases superior) effectiveness of substrate modification. One noteworthy example is the VISTA (Ablation of Clinical Ventricular Tachycardia Versus Addition of Substrate Ablation on the Long Term Success Rate of VT Ablation) trial of Di Biase et al,^14^ who studied 118 patients with ICM-related VT randomized to conventional VT induction and mapping or a substrate modification technique called scar homogenization. At 1 year of follow-up, patients in the substrate modification group had lower rates of both VT recurrence (15.5% vs 48.3%, *P* <.001) and mortality or rehospitalization (20.7% vs 46.7%, *P* = .003) compared to those in the conventional VT mapping group.^14^ Two subsequent meta-analyses, which included patients with NICM as well as ICM, reached more modest conclusions, indicating a nonsignificant trend of lower all-cause mortality and VT recurrence with substrate modification compared to VT induction and mapping.^13^^,^^15^

Yet even with modern substrate modification approaches, attempting to induce VT immediately after ablation remains standard practice. This practice stems from the fact that inducibility of VT after ablation has been associated with a higher risk of VT recurrence and mortality in previous studies.^21^, ^22^, ^23^ In some cases, operators may tailor their strategy based on the results of postablation VT induction. For example, if VT is inducible following endocardial substrate modification, VT mapping and/or epicardial access may be pursued. Nonetheless, the predictive value of VT inducibility after ablation seems to be only modest. With respect to VT recurrence or death at 1 year in 298 patients following VT ablation, Oloriz et al^21^ found that VT inducibility had a negative predictive value of 71% and a positive predictive value of only 43%. Tung et al^22^ showed that the VT recurrence rate among 1277 noninducible patients followed for a median of 17 months after ablation was 58%. Furthermore, the extent to which additional ablation based on positive inducibility testing improves clinical outcomes remains unproven, as to date there have been no randomized trials comparing the inclusion vs omission of inducibility testing after VT ablation. In our study, attempted VT induction during ablation did not lower the risk of VT recurrence or the composite of VT recurrence, transplant, or death during long-term follow-up. Moreover, the subgroup of 22 patients who had VT induction attempted at the end of ablation (ie, inducibility testing) experienced VT recurrence at a rate similar to the remainder of our patient cohort. These findings support our previously described schema for VT ablation risk stratification,^18^ which suggests avoiding VT induction in patients at highest risk for periprocedural hemodynamic compromise (PAINESD score ≥15 and/or with known unstable VT).

Finally, we found that NICM independently predicted a higher incidence of VT recurrence at 1 year compared to ICM. This association has been described previously and may reflect the more complex scar geometry often seen in NICM patients with VT.^22^^,^^24^

### Study limitations

Our results should be viewed as hypothesis-generating in light of our study’s small sample size and retrospective, nonrandomized design. Residual confounders may be present despite our attempts to control for participants’ baseline characteristics. For example, we did not adjudicate “acute procedural success” as a function of complete or incomplete substrate modification. Although we have no reason *a priori* to suspect an uneven distribution of acute procedural success between induction groups, such an uneven distribution could bias our results if present. One could speculate that our findings of equivalent long-term efficacy outcomes represent type II error, and that the conventional strategy of VT induction is, in fact, superior to noninduction. However, we believe this is unlikely given the statistically greater VT burden reduction and the numerically lower incidence of VT recurrence and VT recurrence, heart transplant, or death in the noninduction group. These trends remained consistent when comparing the absence vs presence of any intraprocedural VT (regardless of induction strategy). Furthermore, the use of activation/entrainment mapping was nonsignificantly associated with a *higher* rate of VT recurrence (SHR 1.45, 95% CI 0.57–3.71). Therefore, our findings of comparable long-term efficacy cannot be attributed to “crossover” (ie, activation/entrainment mapping of spontaneous VT in some patients in the noninduction group and the absence of mappable VT in some patients in the induction group). As noted earlier, our null result with respect to short-term safety outcomes was vulnerable to type II error and confounding by indication, thus highlighting the need for an adequately powered randomized trial to compare induction strategies more definitively. Finally, a more quantitative assessment of heart rhythm and blood pressure during ablation (eg, duration of VT, duration of hypotension) was not available but may be helpful in future analyses to gauge the impact of VT induction on hemodynamic status with greater precision.

## Conclusion

In a single-center retrospective study of 89 patients with structural heart disease undergoing catheter ablation for VT, we demonstrated no difference in the rate of VT recurrence, heart transplant, or death at 1 year of follow-up among patients with or without attempted intraprocedural VT induction. Our findings support the idea that VT induction may be avoided altogether when ablating patients at high risk for hemodynamic decompensation, without sacrificing long-term procedural efficacy.
