# Supplementary material for: Clinical outcomes after ventricular tachycardia ablation with or without induction
Source: Heart Rhythm O2. 2024 Nov 17;6(2):214–23. doi: 10.1016/j.hroo.2024.10.023 (PMC11993795; doi:10.1016/j.hroo.2024.10.023)
Supplement: Supplementary Tables and Figures [file mmc1.docx]

**Supplementary Tables and Figures**

Supplementary Table 1: VT Induction Details


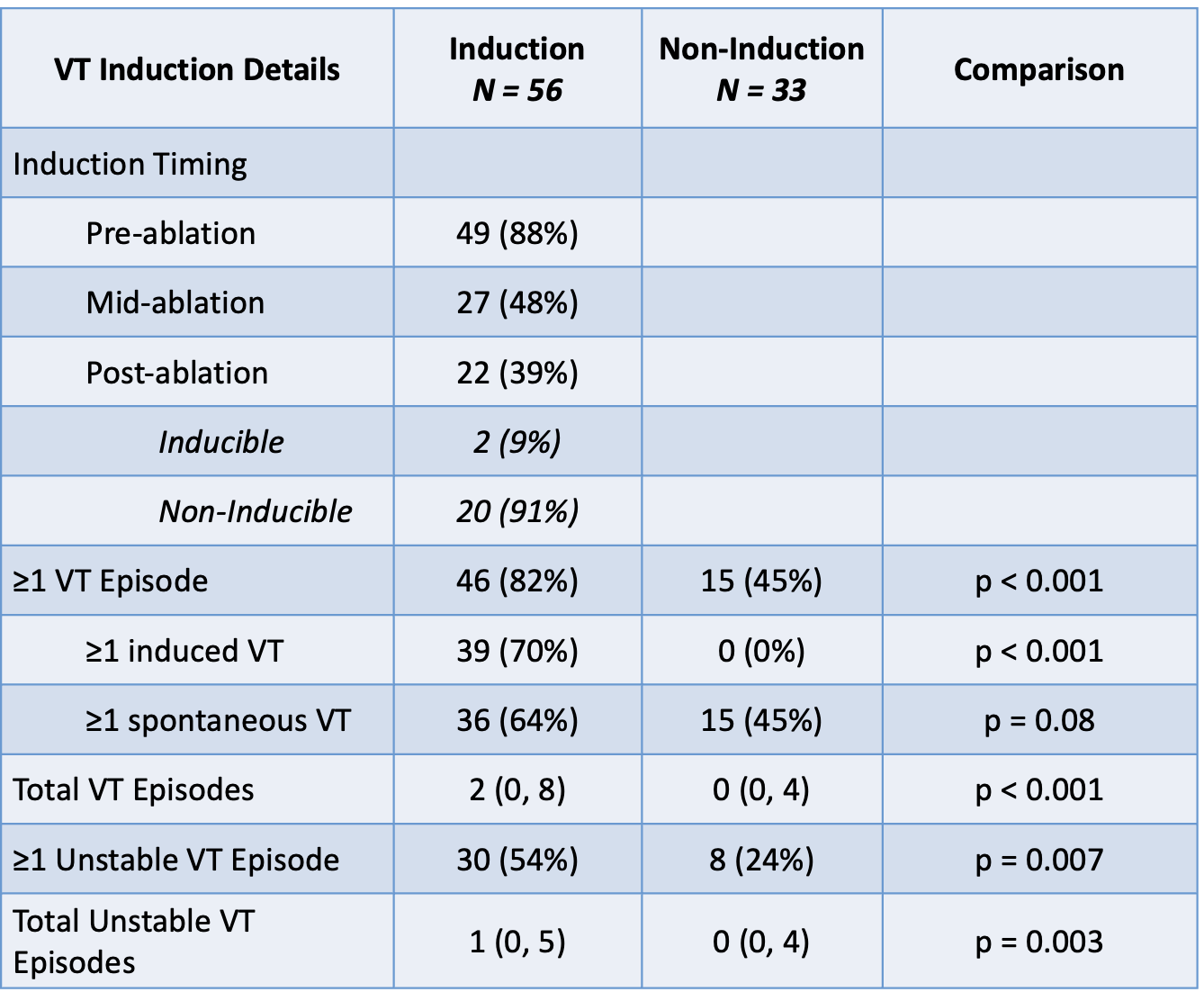


Continuous variables are expressed as median (minimum value, maximum value). VT inducibility post-ablation (italicized above) is expressed as a percentage of the 22 patients in whom post-ablation VT induction was attempted. The above-mentioned VT episodes include both induced and spontaneous VT.

Supplementary Table 2: Acute Procedural Complications


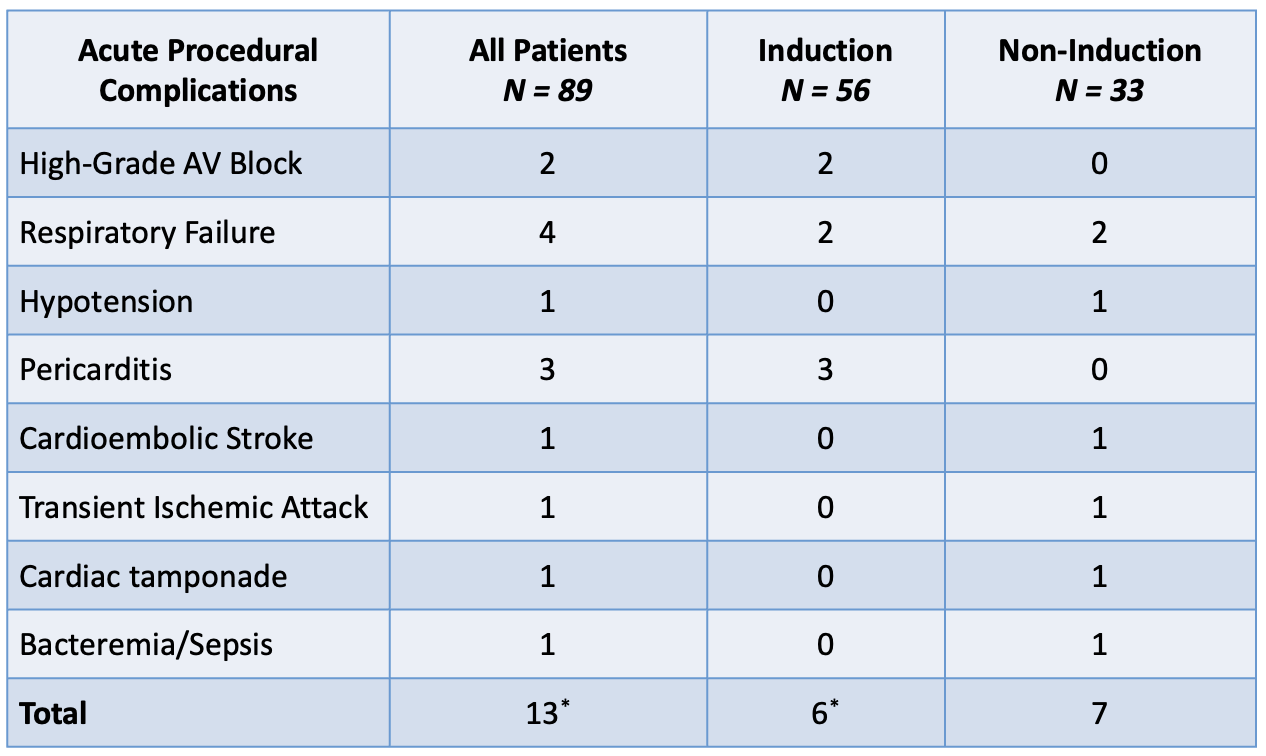


Complications are expressed as raw numbers. ^*^Six patients in the VT induction group experienced a cumulative 7 complications, because 1 patient experienced both high-grade AV block and respiratory failure. Thus, there were a total of 14 complications among 13 patients.

Supplementary Table 3: Clinical Outcomes by Presence or Absence of Intraprocedural VT


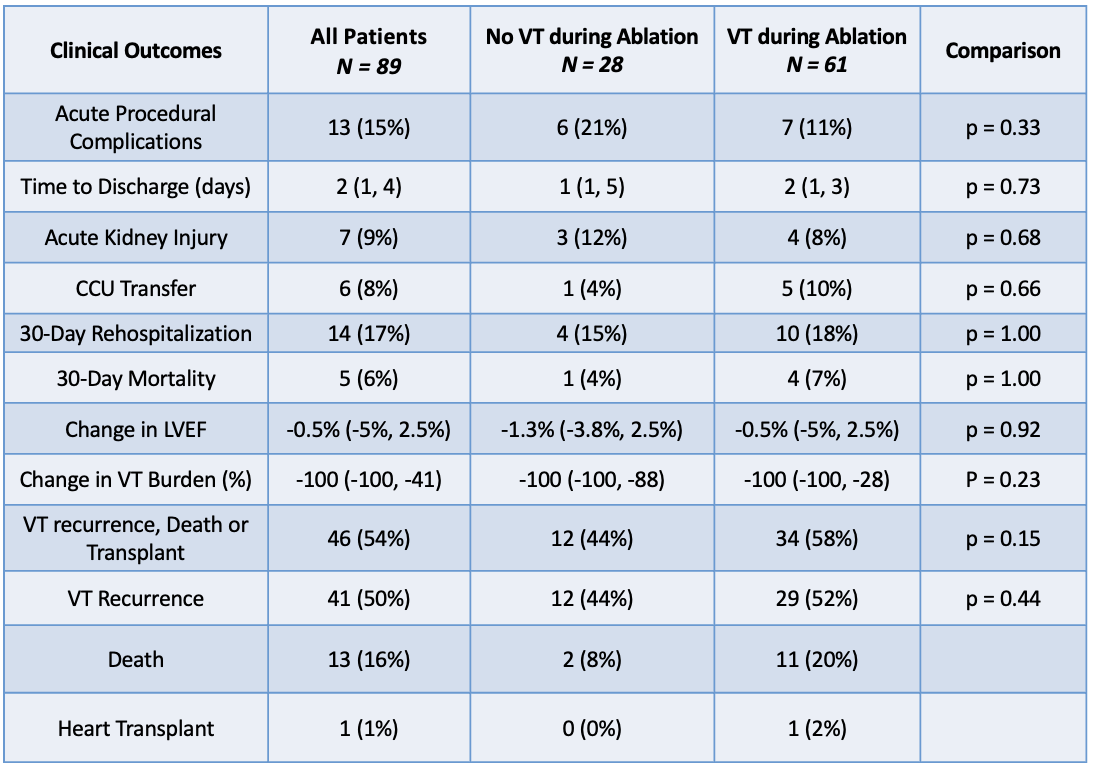


Data was unavailable in the “No VT During Ablation” and “VT During Ablation” groups, respectively, in 1 and 3 patients for time to discharge, 3 and 9 patients for AKI, 3 and 13 patients for CCU transfer, 1 and 4 patients for 30-day rehospitalization, 20 and 43 patients for change in LVEF, and 5 and 21 patients for change in VT burden. Change in LVEF is expressed as an absolute difference. VT recurrence, death, heart transplant, and their composite are expressed as raw number (1-year cumulative incidence). P values for VT recurrence and VT recurrence, death or transplant are derived from univariable competing risks and Cox regression models, respectively.

Supplementary Table 4: Multivariable Competing Risks Regression of VT Recurrence


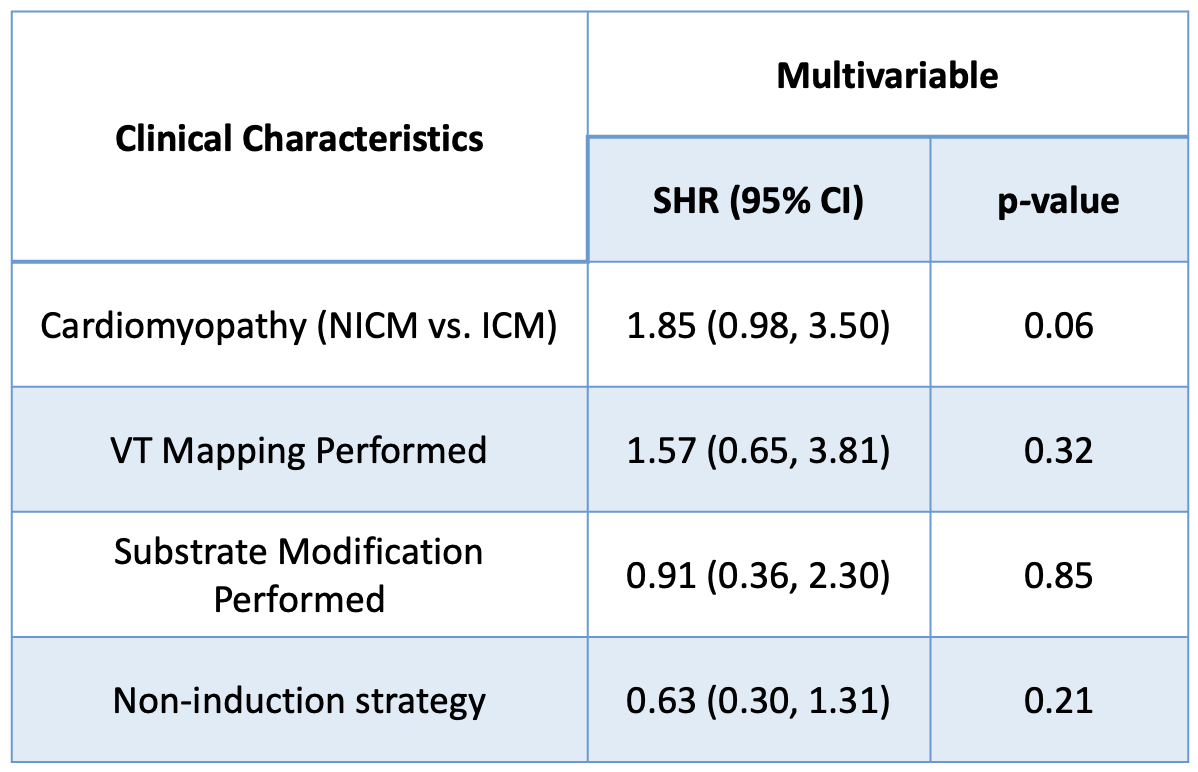


This multivariable regression was performed to assess the possibility that the lower prevalence of NICM and the greater use of substrate modification in the non-induction group may have masked a true benefit of VT induction. SHR, subhazard ratio.

Supplementary Table 5: Multivariable Cox Regression of VT Recurrence, Death or Transplant


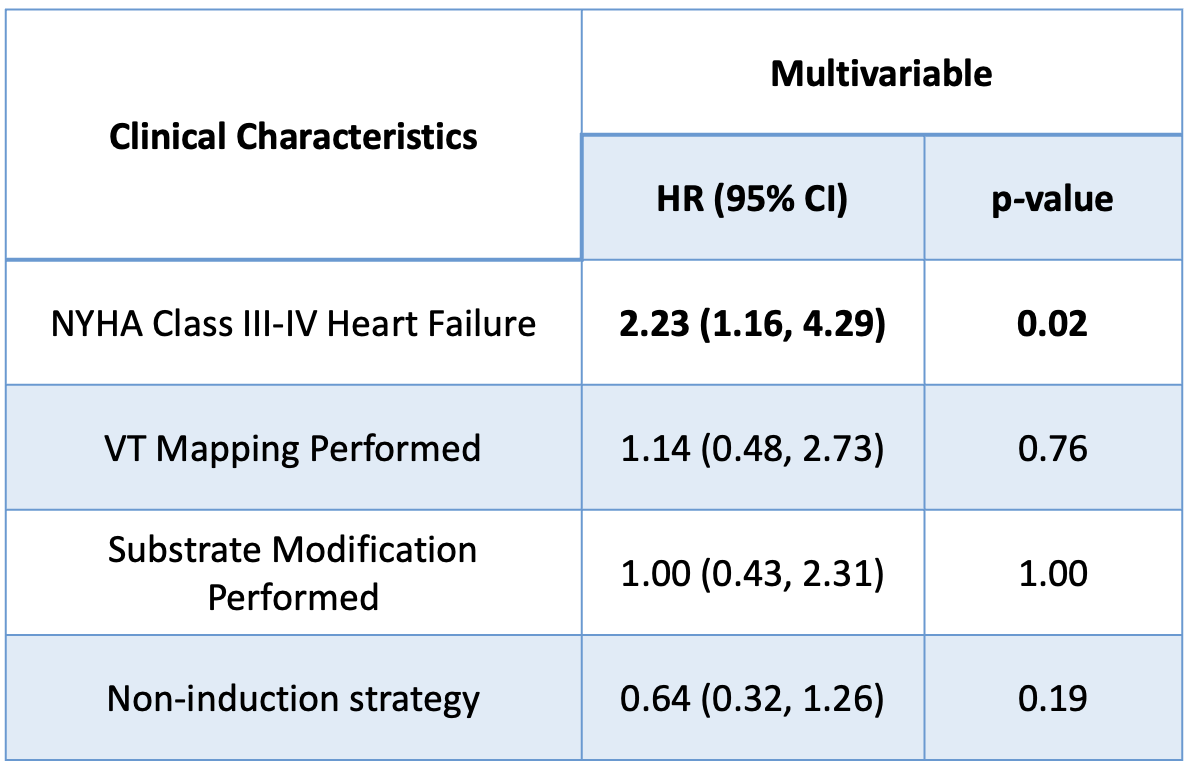


This multivariable regression was performed to assess the possibility that the lower prevalence of NYHA Class III-IV heart failure and greater use of substrate modification in the non-induction group may have masked a true benefit of VT induction. HR, hazard ratio.


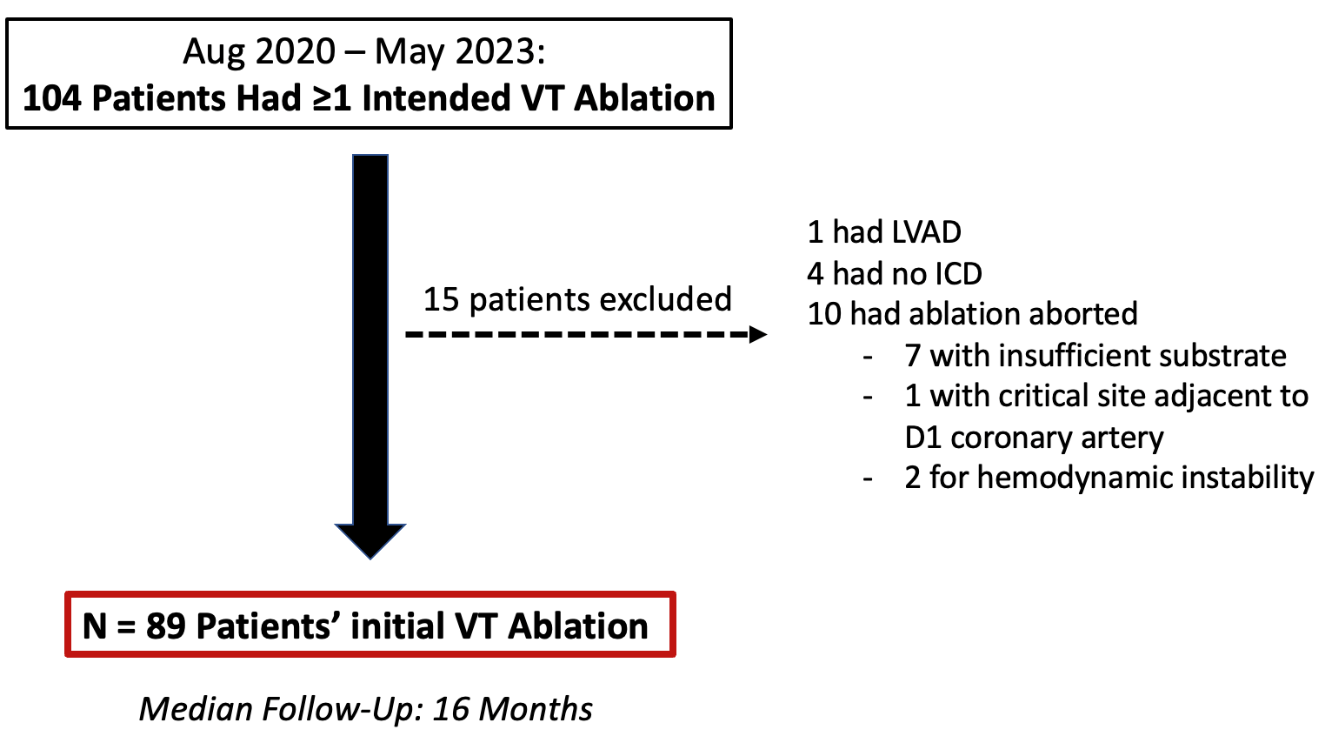


**Supplementary Figure 1: Flow Diagram of Study Design**


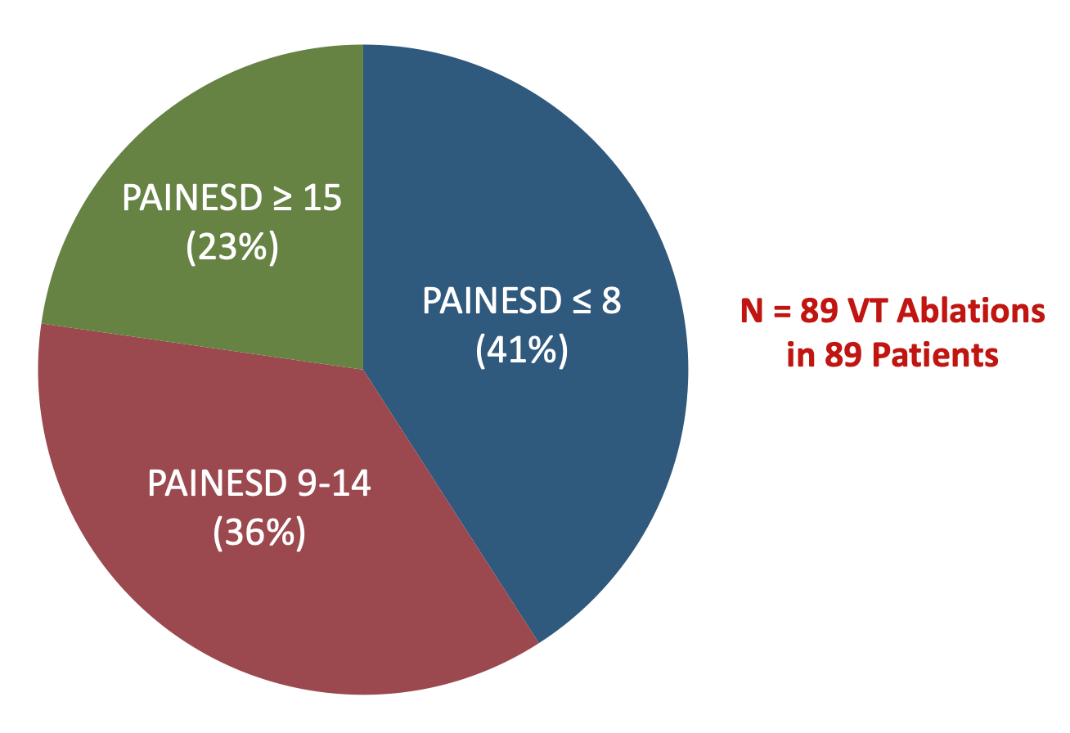


**Supplementary Figure 2: Risk of Periprocedural Hemodynamic Decompensation.** The pie chart above shows the distribution of patients in our study who fell into low- (PAINESD ≤ 8**),** intermediate- (PAINESD 9-14), and high-risk (PAINESD ≥ 15) categories for periprocedural hemodynamic decompensation. Santangeli et al. showed the likelihood of hemodynamic compromise in these risk tertiles to be 1%, 6%, and 24%, respectively. The PAINESD risk score ranges from 0 to 31 points, assigning 5 points for COPD, 3 points for age > 60 years, 6 points for ICM, 6 points for NYHA Class III-IV heart failure, 3 points for LVEF < 25%, 5 points for VT storm, and 3 points for diabetes.


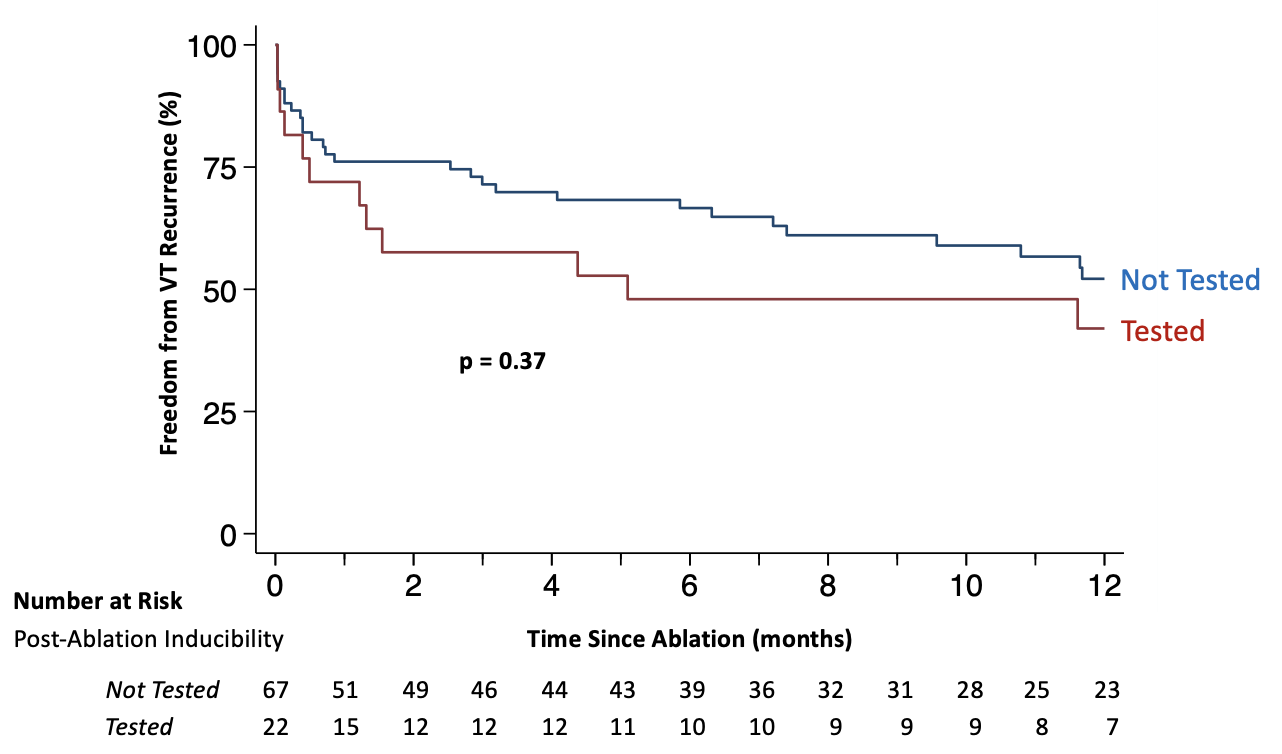


**Supplementary Figure 3: VT Recurrence Stratified by Presence or Absence of Post-Ablation Inducibility Testing.** The cumulative probability of freedom from first recurrent sustained VT is plotted as a function of time elapsed since VT ablation. The p value for absence vs. presence of inducibility testing is derived from a univariable competing risks regression of VT recurrence.
